# First Report of Rubber Collection Bowls & Plastic and Bamboo Water Containers as the Major Breeding Source of *Ae. albopictus* with the Indigenous Transmission of Dengue and Chikungunya in Rural Forested Malaria-Endemic Villages of Dhalai District, Tripura, India: The Importance of Molecular Identification

**DOI:** 10.3390/biomedicines11082186

**Published:** 2023-08-03

**Authors:** Saurav Biswas, Jadab Rajkonwar, Tulika Nirmolia, Sasmita Rani Jena, Ujjal Sarkar, Dibya Ranjan Bhattacharyya, Biswajyoti Borkakoty, Apoorva Pandey, Sarala K. Subbarao, Tapan Majumder, Rocky Pebam, Phiroz Gogoi, Kongkon Mahanta, Kanwar Narain, Ipsita Pal Bhowmick

**Affiliations:** 1 Regional Medical Research Center, Northeast Region (RMRC-NE)-ICMR, Dibrugarh 786001, India; saurav.icmrrmrcne@gmail.com (S.B.); jjrajkonwar22@gmail.com (J.R.); tulikanirmolia@gmail.com (T.N.); ujjalsarkar188@gmail.com (U.S.); biswaborkakoty@gmail.com (B.B.); phirozgogoi@gmail.com (P.G.); kongkonmahanta@gmail.com (K.M.); knarain.rmrcne@gov.in (K.N.); 2 Regional Office of Health and Family Welfare, Kolkata 700106, India; sjena4@gmail.com; 3 Indian Council of Medical Research (ICMR), Ramalingaswami Bhavan, Delhi 110029, India; apoorva.icmr@gmail.com; 4 National Institute of Malaria Research, ICMR, Delhi 110077, India; 5 Department of Microbiology & VRDL, Agartala Government Medical College, Agartala 799006, India; drtapan1960@gmail.com; 6 North East Space Application Centre (NESAC), Department of Space, Government of India, Umiam 793103, India; rocky.pebam@gmail.com

**Keywords:** *Ae. Albopictus*, rubber collection bowls, ponds, water containers, dengue, chikungunya, Tripura

## Abstract

Background: With the reports of indigenous cases of dengue and chikungunya in the forest-covered rural tribal malaria-endemic villages of Dhalai District, Tripura, India, an exploratory study was undertaken to identify the vector breeding sites. Methods: From June 2021 to August 2022, mosquito larvae were collected from both natural and artificial sources in the villages, house premises, and their nearby forested areas outside of the houses. Other than morphological characterisation, *Aedes* species were confirmed by polymerase chain reaction targeting both nuclear (ITS2) and mitochondrial genes (COI) followed by bidirectional Sanger sequencing. Results: *Aedes albopictus* was abundantly found in this area in both natural and artificial containers, whereas *Ae. aegypti* was absent. Among the breeding sources of molecularly confirmed *Ae. albopictus* species, rubber collection bowls were found to be a breeding source reported for the first time. Plastic and indigenously made bamboo–polythene containers for storing supply water and harvesting rainwater in the villages with a shortage of water were found to be other major breeding sources, which calls for specific vector control strategies. Natural sources like ponds and rainwater collected on *Tectona grandis* leaves and *Colocasia axil* were also found to harbour the breeding, along with other commonly found sources like bamboo stumps and tree holes. No artificial containers as a breeding source were found inside the houses. Mixed breeding was observed in many containers with other *Aedes* and other mosquito species, necessitating molecular identification. We report six haplotypes in this study, among which two are reported for the first time. However, *Aedes aegypti* was not found in the area. Additionally, rubber collection bowls, ponds, and water containers also showed the presence of *Culex quinquefasciatus* and *Culex vishnui*, known JE vectors from this area, and reported JE cases as well. Different *Anopheles* vector spp. from this known malaria-endemic area were also found, corroborating this area as a hotbed of several vectors and vector-borne diseases. Conclusions: This study, for the first time, reports the breeding sources of *Aedes albopictus* in the forested areas of Tripura, with rubber collection bowls and large water storage containers as major sources. Also, for the first time, this study reports the molecular characterisation of the *Ae. albopictus* species of Tripura, elucidating the limitations of morphological identification and highlighting the importance of molecular studies for designing appropriate vector control strategies. The study also reports the co-breeding of JE and malaria vectors for the first time in the area reporting these vector-borne diseases.

## 1. Introduction

Indigenous cases of vector-borne diseases such as dengue and chikungunya have been recently reported from malaria-endemic rural areas of the Dhalai district of Tripura, northeast India, and the detection of cases in the dry winter months suggests the existence of perennial breeding sites for vectors [1]. Although not ideal for collecting *Aedes* mosquitoes, CDC light trap collection in the area primarily aimed to collect *Anopheles* species from dusk to dawn and often collected several *Aedes* species, except the established vector species of dengue and chikungunya, *Ae. aegypti* and *Ae. albopictus* [1]. To date, these areas have yet to be studied for detailed information about vectors. Previously, only a few studies reported the presence of *Ae. albopictus* and *Ae. aegypti* in Tripura [2,3], but none from the forested rural areas [4].

There is also no report on the molecular characterisation of *Aedes* species from Tripura. The known vectors *Ae. aegypti* and *Ae. albopictus* belong to the genus *Aedes* and subgenus *Stegomyia*, which contain as many as 132 species [5]. While identifying species within the subgenus *Stegomyia* is often based on morphological features and, in particular for adults, on patterns on the thorax (especially the scutum) and tarsi, there are several limitations of this morphological identification. These morphological characteristics are insufficient to distinguish some species, which may lead to the misidentification of individuals collected in the field. This is a particularly valid concern for *Ae. albopictus* sibling species also mentioned as members of the *Scutellaris* group and *Ae. albopictus* subgroup, as all of these species have quite similar morphological characteristics at the adult (female) stages [6,7,8], including the possibility of occurrence of other isomorphic members of the *Scutellaris* group in the forest areas that are almost unidentifiable from *Ae. albopictus* as adult females [9,10].

The fact that some of these species are sympatric in nature [6,8,11,12] adds to the difficulty in distinguishing between them. Hence, molecular identification serves as a better confirmatory tool for the exact species identification. Moreover, molecular identification not only helps in the genetic characterisation of the species, but also helps to mark the breeding habitats of different species accurately. Furthermore, in the case of mixed breeding in any habitat, as it is challenging to distinguish immature stages of different species, molecular characterisation helps in accurate species identification.

Mitochondrial genes such as cytochrome c oxidase subunit 1 (cox1 or COI) are reported as the most conserved genes in terms of amino acid sequences, have maternal lineages that lack recombination and introns, and are widely used in taxonomic studies [13,14]. Since the COI gene is species-specific, it is a widely used molecular marker to characterise unknown mosquito species and identify sibling species where morphological identification fails to discriminate between mosquito species [15]. A pilot larval survey was undertaken to ascertain the presence of *Aedes* species in the area to identify the types of breeding habitats available in the area supporting the breeding of the known vectors, which can result in the indigenous transmission of the diseases.

## 2. Materials and Methods

### 2.1. Study Area

Dhalai district, located in the northeastern region of Tripura state, is the largest district, with an area of 2313 km^2^. The district is mostly located between two hills, and more than 70% of the land is hilly, with minor streams and woodland. The district’s tropical climate has hot and humid summers, mild winters, and a long rainy season. This study involved the collection from different villages, namely, Tamarai, Ranasai, and Khusidhan (under Karnamani Subcentre (SC)), Satiram, Dongkarai, Ananta Maniya-1, Lakhindra and Forest village (Shikaribari SC), Tilak kumar, Dhansing, Bidhyapara and Khajendra roja par (under Gurudhan SC), Tarjapara and Malda-1 (under Maladapara SC) of Ambassa, and Ganganagar PHC of Dhalai District.

### 2.2. Sample Collection and Diagnosis of Dengue, Chikungunya

The diagnostic test protocol was described in Bhowmick IP et al. (2022) [1]. Briefly, venous blood was collected from the febrile patients, centrifugation was carried out at Ambassa PHC to separate the serum, and it was stored at −20 °C. The sera were sent for testing to the Viral Diagnostic Disease Laboratory (VRDL), Agartala and VRDL, Dibrugarh, where viral testing for dengue and chikungunya was conducted by IgM antibody ELISA (NIV IgM Capture ELISA kits) and the dengue NS1 antigen as per the manufacturer’s instructions. Additionally, the samples were also verified at the ICMR National Institute of Virology, Pune, as per the protocol. Informed consent was obtained from all of the patients. The study protocol was approved by the Institutional Ethical Review Committee on 9 March 2018 (RMRC/Dib/IEC (Human) 2017-18/3573), which is in accordance with the Declaration of Helsinki.

### 2.3. Larval Survey

Larvae were surveyed in different natural as well as artificial sources, inside the house, in house premises (peridomestic areas), and nearby forested areas outside the houses of the villages from June 2021 to August 2022. Collected larvae were transported to the field laboratory and kept with proper labelling for the emergence of the adult. In case of any mortality during rearing, dead larvae were collected and preserved for molecular species identification and characterisation.

### 2.4. Adult Collection

Although the light trap is unsuitable for *Aedes* collection, a few daytime light traps were installed outdoors in two rubber gardens and forest areas.

Also, the dusk-to-dawn CDC light traps set for the routine entomological surveillance of malaria vector data were checked for the presence of different *Aedes* species.

### 2.5. Morphological Identification of Mosquito Species

Adults emerging from the collected larvae were first morphologically identified to species level following the standard keys [6,7] and later subjected to molecular methods to confirm the species.

### 2.6. Molecular Identification of Mosquito Species

Genomic DNA was extracted from individual larvae and the legs of adult mosquitoes. Larvae and adult mosquitoes emerging from different types of containers were processed for molecular identification. Briefly, either one leg of an individual mosquito sample was excised, or the whole larva was taken, maintaining sterile conditions, and was placed in a 1.5 mL microcentrifuge tube. Leg and the larva were then ground individually with a sterile pestle. Further extraction was performed following the QIAamp DNA mini kit protocol (Qiagen, Germantown, CA, USA). Finally, DNA was eluted in 35 μL nuclease-free water and stored at −20 °C for further molecular analysis. For molecular identification of the mosquito species, polymerase chain reaction (PCR) was carried out targeting the mitochondrial cytochrome oxidase region I (COI) and Internal Transcribed Spacer Region II (ITS2) of nuclear DNA as previously described by Kumar et al. [16] and Walton et al. [17]. PCR was performed for all collected dead larvae separately (except for the cases where they decayed in the water) and for the adults which emerged from each type of container, and of those, 12 were sequenced. All the PCR reactions were carried out in 50 μL reaction volume containing 2X Promega master mix with 5 μL DNA template in leg DNA and 2 μL in larval DNA. A known *Ae. albopictus* DNA sample was taken as the positive control, and nuclease-free water was used as a negative control.

PCR products were then analysed on 1.5% agarose gel stained with 0.5 μg/mL ethidium bromide solution under a UV transilluminator (BioRad XR). The amplified products were then gel purified following the manufacturer’s protocol of Wizard Gel and PCR Clean-Up Systems (Promega, Madison, WI, USA). The amplicon size of the COI region was approximately 720 bp for all the samples, and for the ITS2 region, the amplicon size was 450–600 bp, depending upon the mosquito species. For *Ae. albopictus* ITS2, the PCR amplicon size was 600 bp.

### 2.7. Sequencing of ITS2 and COI Gene and Phylogenetic Analysis

To confirm the mosquito species, a selected number of PCR cleaned-up COI and ITS2 PCR products were outsourced for bi-directional Sanger sequencing. Sequences were edited and trimmed using Bio-edit v7.0.5.3 software [18]. The ten edited sequences of COI and two of ITS2 were submitted to the GenBank database. BLAST similarity search was also performed using the NCBI database [19], and available similar global sequences were downloaded to construct a phylogenetic tree in MEGA X software [16]. The neighbour-joining phylogenetic tree was constructed for the COI gene by the Maximum Likelihood statistical method, applying the Tamura-3 parameter model by using a discrete Gamma distribution (+G) with five rate categories with 1000 bootstrap values [19,20] after conducting the best model test in MEGA X. The total number of haplotype and haplotype diversity, the number of polymorphic sites, and the average nucleotide diversity among the isolates of this study were calculated by DnaSP v.6 [21]. Tajima’s D and Fu and Li’s D and F neutrality tests were also performed in DnaSP v.6, with a computing sliding window length of 100 sites and step size of 25 sites.

### 2.8. Haplotype Network Analysis

Haplotype network analysis was carried out by PopART v.1.7 software [22], applying the Minimum Spanning method for the COI gene. COI gene sequences of the global *Ae. albopictus* population were downloaded from GenBank, NCBI database [23]. A total of 110 COI sequences (including ten sequences of this study) of *Ae. albopictus* were considered for haplotype network analysis.

### 2.9. Preparation of Ecological Maps with the Cases and Vectors

Land use land cover (LULC) mapping was prepared using the ortho-rectified Indian Remote Sensing satellite data, Cartosat-1 (2.5 m) and LISS-IV (5.8 m), employing on-screen visual interpretation techniques in the Geographical Information System (GIS) platform. Major LULC categories and subcategories were delineated and updated using the latest data (2019) on the spatial layer, initially prepared under NRSC/ISRO’s Space-based Information Support at 1:10,000 scales. In addition, field verifications were made by the project team to check the accuracy of the interpreted data.

The geolocations of the houses found positive for dengue and chikungunya and the container locations found positive for *Ae. albopictus* were plotted on the LULC maps.

## 3. Results

### 3.1. Diagnosis of Dengue, Chikungunya

In 2021, 12 cases of dengue, 19 of chikungunya, and 34 having both infections were detected in the study area. The blood serum was collected and examined from fever patients through active and passive community surveillance, along with a few patients admitted to the PHCs.

### 3.2. Larval Survey

Of the 306 containers surveyed for larvae, 59 (19.28%) were found positive for the genus *Culex*, 80 (26.14%) for *Aedes*, and 9 (2.5%) for *Culex*, *Aedes*, and *Armigeres*, as shown in Table 1. A total of 158 (51.6%) containers were found negative. In the rubber collection bowls, *Aedes*, *Culex*, and *Armigeres* species were breeding in the association. A few unknown species belonging to the *Scutellaris* group resembling *Ae. albopictus* and *Aedes iyengari* belonging to the *Diceromyia* subgenus breeding was also found in association with *Ae. albopictus* (RMRC unpublished data). A total of 218 *Aedes* larvae were collected; out of that, 180 emerged as adults. One *Ae. albopictus* sample was caught by the light trap. A total of 108 *Culex* larvae were collected, and 70 *Culex* emerged. *Culex* was identified morphologically, which includes *Culex quinquefasciatus*, *Culex vishnui*, and *Culex pseudovishnui*.

**Figure 1:**
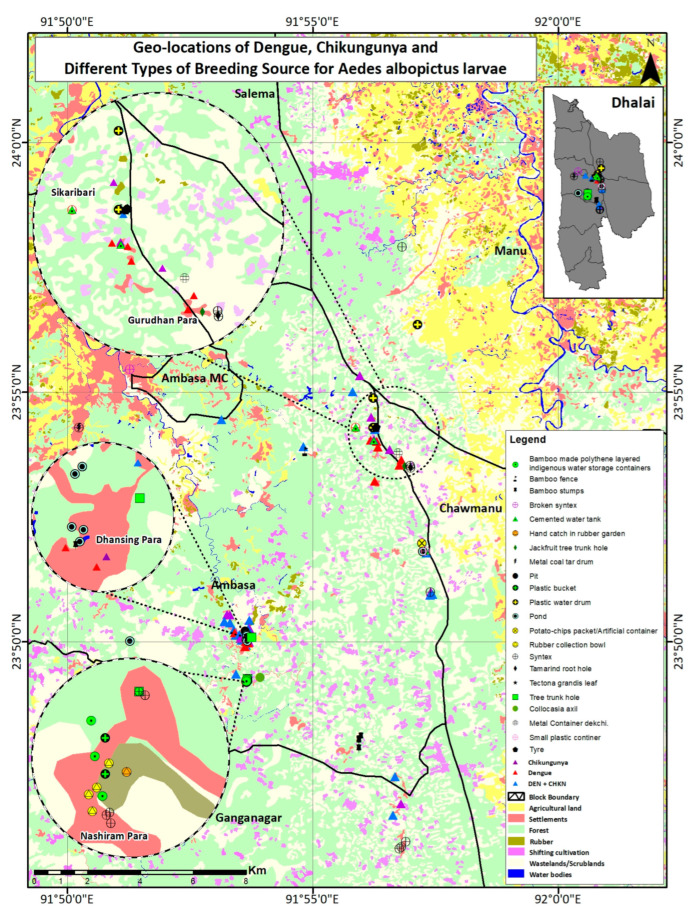
LULC map showing the geolocation of the chikungunya and dengue cases along with the positive container locations. Some areas are zoomed in to show the nearby rubber garden and forested area.

The LULC map shows the geolocation of the cases along with the positive container locations (Figure 1), where the proximity of the study areas to the forest can be seen for most of the collections.

**Figure 2:**
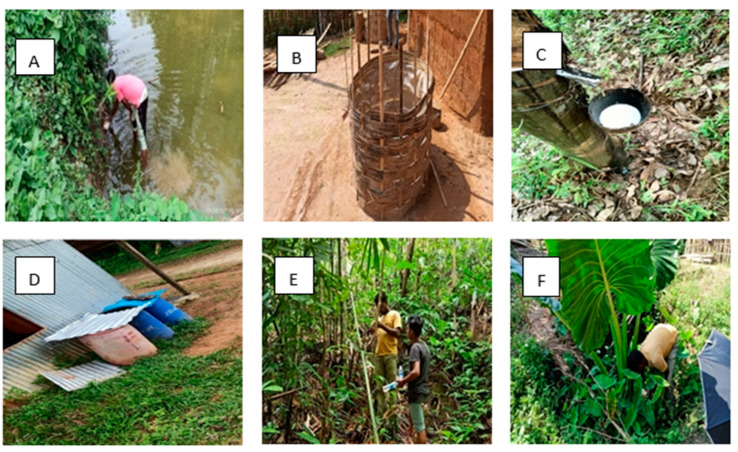
Different natural and artificial containers as breeding sources for *Ae. albopictus.* (**A**) Pond; (**B**) special bamboo structure lined inside with polythene; (**C**) rubber collection bowl; (**D**) artificial container; (**E**) bamboo stumps; and (**F**) *Colocasia* axil.

As shown in Table 2 and Figure 2, the survey revealed that water storage plastic tanks made by the company Sintex, usually of a 500–1000 litre capacity, other smaller water storage containers, indigenously made bamboo tanks with polyethene sheets inside, rubber collection bowls, and plastic buckets are the major breeding source of *Ae. albopictus* in the area. Some of the images of the larval breeding ground of *Ae. albopictus* are shown in Figure 2. Apart from these containers, *Ae. albopictus* breeding was also reported in ponds, *Tectona grandis* leaves that fell on the forest floor containing a small amount of rainwater, *Colocasia* axil, and pits on the ground. Breeding of *Ae. albopictus* was observed in both clear and turbid water, small containers, and large water bodies like ponds, as shown in Table 1. The percentage positivity rate of different types of containers for *Ae. albopictus* is shown in Figure 2.

Rubber bowl collections containing *Ae. albopictus* were also found to harbour *Aedes iyengari* larvae. No *Aedes aegypti* was found in this study from the study area. There were few water containers inside the houses, and none of them were found to contain larvae. They were mostly covered and used for drinking and cooking purposes. The household index calculated for August 2022 for Satiram and Ranasai was 7.7% and 17.3%, respectively. The container index of the different containers during the study period is shown in Table 1.

### 3.3. Phylogenetic Analysis

**Figure 3:**
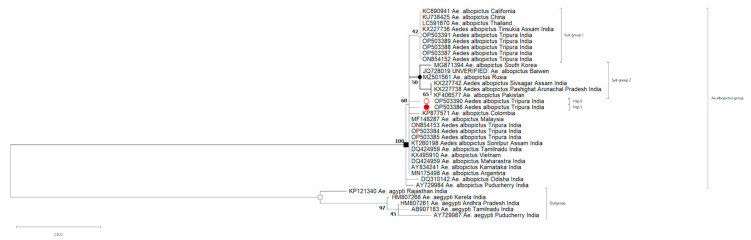
Neighbour-joining phylogenetic (original) tree of *Ae. albopictus* COI gene inferred by using the Maximum Likelihood statistical method and applying the Tamura-3 parameter model with 1000 bootstrap values in MEGA-X. The tree was rooted to *Ae. albopictus* isolates of India.

A total of ten COI and two ITS2 gene sequences of *Ae. albopictus* were generated in this study and were submitted to the GenBank database. The details of the collection sites and containers with the positive isolates are provided in the supplementary Table S1. The phylogenetic analysis of the COI gene was carried out along with other *Ae. albopictus* isolates reported earlier from various parts of India and other parts of the globe. In the phylogenetic tree, the *Aedes albopictus* isolates with accession number ON854152 (Satiram, water drum), OP503387 (Dongkarai, light trap collection), OP503388 (Satiram, light trap collection), OP503389 (Ranasai, light trap collection), and OP503391 (Ranasai, rubber collection bowl) clustered with isolates from India, Thailand, China, and California. The isolates OP503386 (Ranasai, rubber collection bowl, Hap-3) and OP503390 (Ranasai, plastic bucket, Hap-6) formed separate branches in the tree (Figure 3).

The resulting phylogenetic tree delineated the presence of *Ae. albopictus* mosquito species, which were collected from artificial water containers like Sintex water tanks, plastic buckets, rubber collection bowls, chips packets, and natural containers such as ponds as habitats in Dhalai, Tripura, of Northeast India (Figure 3). The two sequences of ITS2 also confirmed the presence of *Ae. albopictus* in both artificial (rubber collection bowl) and natural (bamboo stump) as habitats.

### 3.4. Haplotype Network Analysis

**Figure 4:**
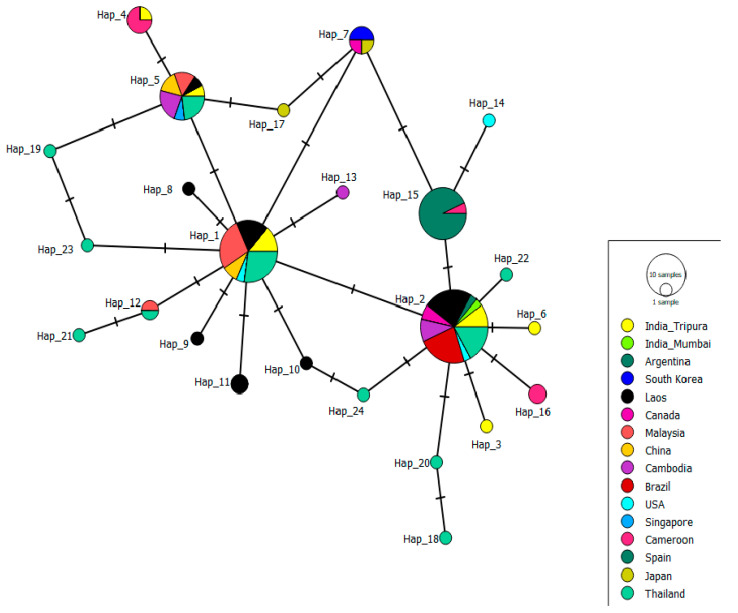
Haplotype network of global *Ae. albopictus* population based on COI gene created using Minimum spanning network. The size of the vortex represents the frequency of the haplotype. Isolates are colour-coded according to the country of origin. The haplotype of the present study is shown in yellow.

Based on the 100 global sequences of *Ae. albopictus*, the COI gene aligned with ten isolates of this study; the total haplotypes calculated was 24. Among the ten isolates of this study, six haplotypes were calculated H-1 (*n* = 3), H-2 (*n* = 3), H-3 (*n* = 1), H-4 (*n* = 1), H-5 (*n* = 1), and H-6 (*n* = 1) (Figure 4). The H-1, H-2, H-4, and H-5 haplotypes have been reported earlier from India and other countries like Laos, Cambodia, Thailand, China, Singapore, Malaysia, Cameroon, USA, and Brazil. Hap-3(OP503386) and Hap-6 (OP503390) are the two new haplotypes observed in this study (Figure 4).

### 3.5. Polymorphism and Population Genetics Analysis of COI Gene in Ae. albopictus

The total number of haplotypes calculated from the isolates of this study was six, and haplotype diversity (Hd) was 0.867. No haplotype diversity was seen among the isolates of Brazil and Spain. The isolates of this study and that of Thailand share a similar haplotype diversity of 0.867 and 0.885, respectively (Supplementary Table S2).

The highest number of haplotypes was observed among the Thailand population (*n* = 11). Nucleotide diversity (Pi) and the average number of nucleotide differences (k) were highest among the Cameroon isolates. For the present study population, Pi and k values were 0.002 and 1.51, respectively, relatively similar to the *Ae. albopictus* isolates of Laos, Thailand, and Cambodia (Supplementary Table S2).

## 4. Discussion

Our study is the first of its kind from Tripura, focusing on detailed molecular and genetic analysis of *Ae. albopictus* and comparing it with different regions of India and the world (Figure 3 and Figure 4; Table S1). The study identified varied natural and artificial breeding habitats of the molecularly confirmed *Ae. albopictus* species (Figure 1 and Figure 2; Table 1 and Table 2). It also reported rubber collection bowls to be a major breeding source in the villages of Ranasaipara under Karnamani Subcentre of Ganganagar Primary Health Centre (PHC) and Satirampara under Shikaribari Subcentre of Ambassa PHC, in Dhalai district, Tripura. Rubber bowls were previously described by only one study from Kerala, India [24]. Even though some earlier studies from India reported plastic drums, containers, buckets, and cemented water tanks as breeding sources of *Aedes* species [25,26], none of these studies were from forested rural areas.

We were able to collect larvae of *Ae. albopictus* from the rubber collection bowl and also the adults using light traps placed in the rubber gardens from 2 to 5 pm, indicative of dark shaded areas of the rubber garden serving as the resting place for *Ae. albopictus*. *Aedes* mosquitoes were also able to be collected by aspirators in the rubber garden area when they attacked people entering the garden. The rubber gardens not only provide a good breeding source for *Ae. albopictus* throughout the year, but adult females also tend to gather there, as evident from the hand and light trap catch, indicating that it can be a good resting place. The mosquitoes collected from the rubber collection bowls were identified as *Ae. albopictus*, by both molecular and morphological methods. *Ae. iyengari*, other *Aedes* sp., and *Culex quinquefasciatus* larvae were also found in the same container. We also came across some dead larvae and instances of mixed breeding, based on which we can say that determining the species based on larval morphology and attributing the breeding source to that particular species can often be erroneous. Hence, rather than conducting studies by pooling samples or relying only on larval morphological identification, molecular identification and breeding habitat analysis of molecularly confirmed species is imperative. It should be noted that, in addition to *Ae. albopictus*, many other species such as *Ae. novalbopictus*, *Ae. patriciae*, *Ae. pseudoalbopictus*, *Ae. subalbopictus*, *Ae. unilineatus*, of the albopictus subgroup and *Ae. krombeini*, *Ae. malayensis*, and *Ae. scutellaris* of the scutellaris subgroup of the genus *Stegomyia* are reported in India [27]. Cryptic species of the *Ae. albopictus* subgroups have recently been reported from China and Vietnam [9,10] and are also suspected in our studies (unpublished data). The presence of similar-looking morphological species reiterates the requirement of molecular identification at both the larval and adult level since the existence of cryptic species can be common [28].

Haplotype network analysis of the COI sequence of *Aedes albopictus* was reported for the first time in Tripura, India. This analysis resulted in the identification of two new haplotypes, Hap-3 (Accession No: OP503386, Ranasai, rubber collection bowl) and Hap-6 (Accession No: OP503390, Ranasai, plastic bucket) from the Dhalai district of Tripura, which was not reported in other parts of the world (Figure 4). Hap-3 and Hap-6 form separate branches in the phylogenetic tree and do not cluster with any subgroup. Although nucleotide differences were observed among the Tripura isolates at some positions, no changes in the amino acid sequence were found.

Our study focuses on the varied breeding habitat of the molecularly confirmed *Ae. albopictus* species and, owing to the ecological plasticity, *Ae. albopictus* is shown to use various man-made containers as its breeding habitat [29]. For the first time, rainwater collected in the *Tectona grandis* leaves fallen in the forest, indigenous water tanks made up of bamboo, and polythene sheets were reported as the breeding habitat of *Ae. albopictus*. The pond, although a common breeding place for some species of *Culex* and Anopheles, was found to be another unusual source for *Ae. albopictus*. Further collections may be required to confirm these water bodies as regular breeding sources for *Ae. albopictus*. In addition, breeding was also observed in bamboo stumps, tree holes, and *Colocasia axils*, which can be categorised as natural breeding sources. *Colocasia axil* is normally found to harbour the breeding of *Malaya* species and, from our studies, may be considered a natural container favouring mixed breeding with *Ae. albopictus*. Our study demonstrated mixed breeding in various types of natural and artificial containers and reasserts the importance of molecular identification. As shown in Table 1, we found breeding in clear, turbid, and coloured water. It is reiterated that we included only those molecularly confirmed samples; hence, some of the samples can be missed where the larvae became dead and decayed in the water. Calculating different indices from the longitudinal study in selected locations is currently underway and can produce important information on the seasonality of the vector abundance. In this study, we obtained *Ae. albopictus* throughout the year (Table 1), as there were different kinds of artificial containers, and even sporadic rain in the non-rainy season months caused water accumulation in the rubber collection bowls when the collection of rubber was not being conducted.

*Ae. albopictus* is not only a vector of the dengue, chikungunya, and zika viruses, but this species is also capable of transmitting 14 other arboviruses [30]. Considering its opportunistic feeding behaviour on a variety of animals, including humans, *Ae. albopictus* has been considered a competent bridge vector for several arboviruses, having the possibility of transmitting such zoonotic viruses to the human population [30]. Tewari et al. (2004) [31] first isolated the dengue virus from *Ae. albopictus* in rural areas of south India. Studies carried out earlier in urban areas of four Northeastern states established *Ae. albopictus* as a dengue vector in this region [32], and this study also reported that *Ae. albopictus* (94% composition) predominates over *Ae. aegypti* in a small township of Arunachal Pradesh, which has good vegetation cover compared to other areas with much less vegetation cover. The present study was in the rural areas of Tripura and *Ae. aegypti* was not encountered.

Additionally, rubber collection bowls, ponds, and water containers showed the presence of *Culex quinquefasciatus* and *Culex vishnui*, known JE vectors from this area. We had previously reported JE infections from the area [1], and during the study period, we also detected several JE mono and mixed infections from the area (unpublished data). This area is known to be highly malaria-endemic, as reported by several studies [33,34,35,36]. *Anopheles vagus* and other unidentified larvae of the genus *Anopheles* were found during this study period, mainly from the ponds (unpublished data). Hence, this study corroborates this area as a hotbed of several vectors and vector-borne diseases.

These study outcomes can have major implications in Tripura as this small state has one of the highest rubber plantations in the country. Even though rubber plantations are not very common in our study district, Dhalai, the findings of our study can be extended to other districts of Tripura like Gomoti, Sepahijala, Unokoti, South district, etc. which have comparatively higher rubber plantations [24] and are at high risk for dengue and chikungunya. In 2021, a dengue outbreak was reported in some areas of the Gomoti, Sepahijala, and Unokoti districts, which mostly adjoin the rubber plantations (unpublished data), reasserting this study’s findings. Indeed, there has been an outbreak in some areas of these districts during October–November 2021, and the role of *Ae. albopictus* as a vector and its relationship with the rubber gardens have been explored (unpublished data).

The findings of the artificial containers, plastic water drums, or indigenously made bamboo tanks with polyethene sheets inside them as major sources of different vector species call for special attention. These are used to store water supplied by the Government and harvest rainwater. As there was almost no piped water supply in the area, the practice of water storage in these containers to avoid water scarcity is rampant. We found that, to facilitate rainwater harvesting, many of these containers are not covered and remain partially or fully open, aiding in the breeding of *Ae. albopictus*. These containers are deep and are often regularly used, but that does not prevent the breeding as only the upper part is used, rendering the larvae at the bottom undisturbed. Special innovative targeted strategies and inter-sectoral coordination are required to stop the breeding in all these containers.

## 5. Conclusions

Given the current findings, an active Information, Education, and Communication (IEC) campaign and particular vector control initiatives in the area are required, especially given the artificial breeding grounds such as plastic water storage containers and rubber collection bowls that are the primary source of *Ae. albopictus* in the area. For in-depth research of the vector species, accurate molecular identification of mosquito species and their sub-species is required, which may add information to the current gene database and aid in investigating species variation among species from different regions of the world.

## Figures and Tables

**Table 1:** Container positive and container index observed in different villages for *Aedes* species.

Place (Village)	Time of Survey	Containers Searched (No.s)	Container Type (Positive) for *Aedes.*	Water Condition	No. of Containers Positive for *Aedes* Species (Morphological and Molecular Identification)	Container Index for *Aedes* Species	Containers Positive for Mosquito Species Other than *Aedes* Species (Morphological Identification)
Tamarai	July 2021	8	Bamboo stump	Coloured, clear	2 *Aedes albopictus*	22.2	3 *Culex* spp. * *Culex*
	May 2022	4	Bamboo stump	Turbid, coloured	1 *Aedes albopictus*		1 *Culex* spp. *
	July 2022	4	Bamboo stump	Coloured	1 *Aedes albopictus*		2 *Culex* spp. *
	August 2022	2	Bamboo stump	Clear	0		0
Satiram	July 2021	6	Water drum	Clear	1 *Aedes albopictus*	38.1	*3 Culex* spp. *
	August 2021	2	Well	Clear	0		1 *Culex* spp. *
	April 2022	6	Water drum and tyre	Clear	3 *Aedes albopictus*		2 *Culex quinquefasciatus*, *Culex* spp. *, 1 mixed with *Culex* spp. * and *Aedes* spp. *
	May 2022	5	Cemented water tank and plastic water drum	Polluted, clear	4 *Aedes albopictus*		1 *Culex* spp. *
	July 2022	5	Coal tar metal drum	Clear, Coloured	4 *Aedes albopictus*		1 *Culex* mixed with *Aedes* spp. * and *Culex* spp. *
			plastic drum				
			Mixed breeding in tyre				
	August 2022	18	Metal coal tar	Clear	4 *Aedes albopictus*		0
			drum and plastic (Sintex) water tank				
Tilak Kumar	June 2021	4	Pond	Clear	0	28.6	1 *Culex* spp. *
	July 2021	4	Chips packet	Turbid	1 *Aedes albopictus*		0

	April 2022	3	Cemented water tank and pond	Turbid	2 *Aedes albopictus*		0
	July 2022	3	Small plastic container	Clear	1 *Aedes albopictus*		0
Ranasai	December 2021	12	Plastic bucket	Clear	3 *Aedes albopictus*	50	1 *Culex* spp. * 1 *Armigeres* spp.*
			Plastic sheet				
	February 2022	1	Sintex water tank	Clear	1 *Aedes albopictus*		0
	April 2022	10	Rubber collection bowl	Clear	3 *Aedes albopictus* (1 mix with *Aedes iyengari*)		1 *Culex quinquefasciatus*
	May 2022	17	Rubber collection bowl	Clear	12 *Aedes albopictus*		0
	June 2022	16	Rubber collection bowl	Clear	4 *Aedes albopictus* (1 mixed with *Aedes* spp. *)		0
	July 2022	7	Sintex water tank	Clear	7 *Aedes albopictus*		0
	August 2022	3	Plastic (Sintex) water tank, *Colocasia* axil	Clear	3 *Aedes albopictus*		0
Donkarai	July 2021	4	Tree hole	Turbid	0	42.1	6 *Culex* spp. *
	August 2021	3	Tree hole	Turbid	0		1 *Culex* spp. *
	November 2021	1	Pond	Clear	0		0
	April 2022	1	Tamarind root hole	Turbid	1 *Aedes albopictus*		0
	May 2022	2	Pond	Clear	0		0
	June 2022	1	Stream pool	Clear	0		0
	July 2022	1	Cemented water tank	Clear	1 *Aedes albopictus*		0
	August 2022	6	Plastic (Sintex) water tank, jackfruit tree trunk hole, and aluminium cooking vessel	Coloured, clear	6		3 *Armigeres* spp. *
Dhansingh	July 2021	2	Pond	Coloured	0	16.5	2 *Culex* spp. *
	October 2021	2	Pond	Turbid	0		0
	December 202021	21	Indigenously made bamboo tanks with polyethene sheets inside	Clean	1 *Aedes albopictus*		0
	January 202022	33	Plastic (Sintex) water tank	Clear	1 *Aedes albopictus*		3 *Culex* spp. *
	February 2022	1	Pond	Turbid	0		1 *Culex* spp. *
	March 2022	1	Pond	Clear	0		1 *Culex* spp. *
	April 2022	6	Pond	Coloured, turbid	6 *Aedes albopictus*		2 *Culex vishnui*
			Groundwater pool	Clear			1 *Anopheles vagus*
	May 2022	8	Tree hole	Clear	1 *Aedes albopictus*		1 *Anopheles* spp. *, 3 *Culex* spp. *
			Bamboo stump				
	June 2022	3	Bamboo stump	Clear	2 *Aedes albopictus*		0
			tree hole				
	July 2022	2	Indigenously made bamboo tanks with polyethene sheets inside	Clear	2 *Aedes albopictus*		0
Forest Village	December 2021	13	*Tectona grandis* leaf	Clear	1 *Aedes albopictus*	5.6	3 *Culex* spp. *
	February 2022	3	Pond	Coloured	0		1 *Culex* spp. *
	April 2022	2	Bottle	Coloured	0		1 *Culex* spp. *
Tarjapara	April 2021	2	Ground pool	Turbid	0	70	1 *Culex* spp. *
	December 2021	4	Plastic (Sintex) water tank	Clear	3 *Aedes albopictus*		0
	July 2022	4	Plastic (Sintex) water tank	Clear	4 *Aedes albopictus*		0
Ananta Maniya-1	August 2021	4	Well	Clear	0	16.7	2 *Culex* spp. *
	June 2022	5	Bamboo fence	Turbid	2 *Aedes albopictus*		3 *Culex* spp. * 2 *Armigeres subalbatus*
	July 2022	3	Pond	Turbid	1 *Aedes* spp. *		1 *Culex* spp. * 1 *Armigeres subalbatus*
Khajendra roja Para	Juyl 2022	6	Plastic (Sintex) water tank	Clear	1 *Aedes albopictus*	16.7	0
Khusidhan	July 2021	4	Pond	Turbid	0	0	3 *Culex* spp. *
	October 2021	4	Pond	Coloured	0		1 *Culex* spp. *
	August 2022	2	Pond	Clear	0		*2 Culex* spp. *
Bidhyapara	June 2022	2	Pond	Coloured	0	0	2 *Culex* spp. *
Malda-1	December 2021	4	Plastic (Sintex) water tank and Discarded tyres	Coloured, Clear	0	0	2 *Culex* spp. *
Lakhindra	November 2021	6	Stream pool	Clear	0	0	*4 Culex* spp. *

spp. *—Genus was morphologically identified for these specimens, but the exact species could not be identified.

**Table 2:** Type of containers searched and positivity rate.

Sl. No	Container Type	Searched	Found Positive	Container Positivity %
1	Bamboo stump	13	6	46
2	Pond	47	6	13
3	Indigenously made bamboo tanks with polyethene sheets inside	3	3	100
4	Pit	2	1	50
5	Plastic Water drum	10	7	70
6	Sintex water tank	45	24	53
7	Metal coal tar drum	8	5	63
8	Tyre	4	2	50
9	Cemented water tank	9	3	33
10	*Tectona grandis* leaf	2	1	50
11	Chips packet	1	1	100
12	Small plastic container	3	2	67
13	Plastic bucket	6	2	33
14	Rubber bowl	40	18	45
15	Tree trunk hole	5	2	40
16	*Colocasia axil*	1	1	100
17	Bamboo fence	2	2	100
18	Tamarind root hole	1	1	100
19	Jackfruit tree trunk	1	1	100
20	Metal utensil	1	1	100

## Data Availability

The data presented in this study are available on request from the corresponding author. The data are not publicly available due to ethical and privacy reasons.

## Supplementary Materials

The following supporting information can be downloaded at: https://www.mdpi.com/article/10.3390/biomedicines11082186/s1. Table S1: Isolates of the study with respect to the container and area. Table S2: Diversity and neutrality indices for *Ae. albopictus* global populations based on COI gene.
