# Supplementary material for: First Report of Rubber Collection Bowls & Plastic and Bamboo Water Containers as the Major Breeding Source of Ae. albopictus with the Indigenous Transmission of Dengue and Chikungunya in Rural Forested Malaria-Endemic Villages of Dhalai District, Tripura, India: The Importance of Molecular Identification
Source: Biomedicines. 2023 Aug 3;11(8):2186. doi: 10.3390/biomedicines11082186 (PMC10452501; doi:10.3390/biomedicines11082186)
Supplement: Supplementary file 1 [file biomedicines-11-02186-s001.zip › Supplementary Table S1.pdf]

**Table S1:** Isolates of the study with respect to the container and area.

| **Accession No:** | **Container** | **Area** |
|---|---|---|
| ON854152 | Water drum | Satiram |
| ON854153 | Chips packet | Tilakkumar |
| OP503384 | Pond | Tilakkumar |
| OP503385 | Rubber collection bowl | Ranasai |
| OP503386 | Rubber collection bowl | Ranasai |
| OP503387 | Light trap collection | Dongkarai |
| OP503388 | Light trap collection | Satiram |
| OP503389 | Light trap collection | Ranasai |
| OP503390 | Plastic bucket | Ranasai |
| OP503391 | Rubber collection bowl | Ranasai |
| ON849338 | Bamboo stump | Tamarai |
| OP503895 | Rubber collection bowl | Ranasai |
