# Supplementary material for: First Report of Rubber Collection Bowls & Plastic and Bamboo Water Containers as the Major Breeding Source of Ae. albopictus with the Indigenous Transmission of Dengue and Chikungunya in Rural Forested Malaria-Endemic Villages of Dhalai District, Tripura, India: The Importance of Molecular Identification
Source: Biomedicines. 2023 Aug 3;11(8):2186. doi: 10.3390/biomedicines11082186 (PMC10452501; doi:10.3390/biomedicines11082186)
Supplement: Supplementary file 1 [file biomedicines-11-02186-s001.zip › Supplementary Table S2.pdf]

Table S2: Diversity and neutrality indices for *Ae. albopictus* global populations based on COI gene (N=number of sequences,H=number of haplotype, Hd=Haplotype diversity, S=number of segregating sites, k=average number of nucleotide differences, Pi=nucleotide diversity per site, NA=not applicable (only 1 haplotype is present) *p=statistically significant. Populations with less than 4 sequence were excluded from this calculation.)

Population	N	S	H	Hd	Pi	K	Tajima's D	Fu Li's D	Fu Li's F
Tripura(This study)	10	5	6	0.867	0.00246	1.511	-0.58152, P > 0.10	-1.511, P > 0.10	-0.74018, P > 0.10
Laos	17	6	7	0.794	0.00196	1.206	-1.07510, P > 0.10	-1.418, P > 0.10	-1.52235, P > 0.10
Thailand	23	9	11	0.885	0.00295	1.810	-0.85358, P > 0.10	-0.242, P > 0.10	-0.49040, P > 0.10
Malaysia	8	2	3	0.607	0.00111	0.679	-0.44794, P > 0.10	-0.149, P > 0.10	-0.23785, P > 0.10
Spain	13	0	1	NA	NA	NA	NA	NA	NA
Brazil	7	0	1	NA	NA	NA	NA	NA	NA
Cameroon	6	5	3	0.733	0.00434	2.667	1.21883, P > 0.10	0.938, P > 0.10	1.06342 P > 0.10
Cambodia	6	3	3	0.733	0.00239	1.467	0.60031 ,P > 0.10	0.510, P > 0.10	0.55663 P > 0.10
China	4	1	2	0.667	0.00109	0.667	1.63299,P > 0.10	1.632, P > 0.10	1.276, P > 0.10
